# Should case management be considered a component of obstetrical interventions for pregnancies at risk of preterm birth?

**DOI:** 10.1016/j.ajog.2022.09.022

**Published:** 2022-09-19

**Authors:** Thomas J. Garite, Tracy A. Manuck

**Affiliations:** Sera Prognostics, Salt Lake City, UT (Dr Garite); University of California Irvine, Irvine, CA (Dr Garite); Division of Maternal-Fetal Medicine, Department of Obstetrics & Gynecology, The University of North Carolina at Chapel Hill, Chapel Hill, NC (Dr Manuck); and Institute for Environmental Health Solutions, Gillings School of Global Public Health, The University of North Carolina at Chapel Hill, Chapel Hill, NC (Dr Manuck).

**Keywords:** case management, case managers, nutrition counseling, prematurity education, preterm birth, psychosocial support, smoking cessation, substance abuse counseling

## Abstract

Preterm birth remains the leading cause of morbidity and mortality among nonanomalous neonates in the United States. Unfortunately, preterm birth rates remain high despite current medical interventions such as progestogen supplementation and cerclage placement. Case management, which encompasses coordinated care aimed at providing a more comprehensive and supportive environment, is a key component in improving health and reducing costs in other areas of medicine. However, it has not made its way into the general lexicon and practice of obstetrical care. Case management intended for decreasing prematurity or ameliorating its consequences may include specialty clinics, social services, coordination of specialty services such as nutrition counseling, home visits or frequent phone calls by specially trained personnel, and other elements described herein. It is not currently included in nor is it advocated for as a recommended prematurity prevention approach in the American College of Obstetricians and Gynecologists or Society for Maternal-Fetal Medicine guidelines for medically indicated or spontaneous preterm birth prevention. Our review of existing evidence finds consistent reductions or trends toward reductions in preterm birth with case management, particularly among individuals with high a priori risk of preterm birth across systematic reviews, metaanalyses, and randomized controlled studies. These findings suggest that case management has substantial potential to improve the environmental, behavioral, social, and psychological factors with patients at risk of preterm birth.

## CONDENSATION

Case management is an underrecognized, potentially important clinical tool in obstetrics. By enhancing patient support and addressing multifactorial prematurity—eassociated risk factors, this approach may improve obstetrical outcomes.

It is hard to imagine that Nicholson Eastman, an obstetrician and gynecologist, would have envisioned a world in the 22nd year of the 21st century, where the rate of preterm birth in the United States has not decreased since he made his oft-quoted 1947 statement (>75 years ago), that “only when the factors causing prematurity are clearly understood can any intelligent attempt at prevention be made.”^1^ Although some inroads have been made in understanding the multifactorial etiologies underlying preterm birth, little progress has been made in developing interventions that have a major impact on most of those who happen to deliver prematurely. Interventions such as progesterone (for individuals who have had a previous preterm birth or develop a short cervix) and cervical cerclage (for those who have a history of cervical insufficiency or midtrimester cervical shortening or dilation) are indicated for only a small percentage of patients at risk of preterm birth, and they do not guarantee a term delivery.^2–6^ Other interventions such as bed rest, omega-3 supplementation, prophylactic tocolytic drugs, progestogen supplementation for arrested preterm labor, home contraction monitoring, and others were initially promising but have since been shown to be ineffective.^7–11^

“Care management” or “care coordination,” among other descriptive monikers, was initially introduced in obstetrics in the mid-1980s as a possible nonpharmacologic approach to improve perinatal outcomes by addressing many of the underlying socioeconomic factors that plague prematurity and other adverse pregnancy outcomes. The Commission for Case Manager Certification defines case management as “a collaborative process that assesses, plans, implements, coordinates, monitors, and evaluates the options and services required to meet the client’s health and human service needs. It is characterized by advocacy, communication, and resource management and promotes quality and cost-effective interventions and outcomes.”^12^ Similarly, the Agency for Healthcare Research and Quality defines care coordination as “the deliberate organization of patient care activities between two or more participants to achieve safer and more efficient care.”^13^ In our review, it was seen that the term “case management” was used more than twice as commonly as care coordination. However, the elements of the programs included considerable overlap and were virtually indistinguishable. Therefore, we shall use the term case management to include care coordination.

Other fields of medicine, including oncology and cardiology, which, like obstetrics, involve multiple providers, medications, and clinic appointments, regularly employ case management programs. Specifically, within such programs, the use of designated “patient navigators” (ie, case managers) and stress reduction programs are proven approaches to improve health outcomes. Research from these fields indicates that incorporation of case managers effectively expands access to health services, improves health outcomes, and increases patient support, particularly among vulnerable populations.^14–17^

Case management has also shown great promise when incorporated into prematurity prevention programs. Despite this, case management has neither become integrated into the general lexicon for obstetricians nor has it been included as a component of prematurity prevention programs described in previous publications from the American College of Obstetricians and Gynecologists (ACOG) or from the Society for Maternal-Fetal Medicine (SMFM), including the SMFM Preterm Birth Tool Kit.^18^

The genesis of case management studies addressing the problem of preterm birth likely emanated from original studies by Creasy et al, who developed a risk scoring system for increased risk of preterm birth.^19^ This became the foundation for the first example of a program that, although not labeled as case management, incorporated several key elements of later case management programs as follows: identifying pregnancies at high risk of preterm birth, educating patients regarding self-detection of early signs of preterm labor, and providing weekly visits to specialized prematurity clinics.^20^ In the first year following the implementation of this program, there was a decreased incidence of preterm delivery. Subsequently, a multicenter randomized controlled trial of 5 centers using this system was conducted but found no difference in the rate of delivery earlier than 37 weeks.^21^

The purpose of this Clinical Opinion is 2-fold: to increase awareness and to review the potential benefits of case management in the prevention of preterm birth. It is not the intent of this document to serve as a systematic review or metaanalysis of this subject, as at least 6 such publications exist currently^22–27^; it is rather to summarize their conclusions and describe the interventions used and various outcomes described in 29 individual studies^20,28–55^ that we also reviewed. To identify additional peer-reviewed studies of case management and preterm birth, we searched the literature for and included all articles with the following terms: case management, care management, care coordination (in association with prematurity), prematurity prevention clinics, prematurity clinics, and prematurity prevention programs. Articles focusing on multiple approaches aimed at prematurity prevention were included. Manuscripts focusing primarily on pharmacologic interventions without specific attention to nonpharmacologic interventions were excluded. We neither found case management studies aimed specifically at multiple gestations nor any reports with a substantial number of multiple gestations included in their analyses. Thus, we limited this discussion to studies focused on singleton gestations.

The elements of case management programs described in the included publications are listed in Table 1. Although presented somewhat vaguely in this table, the summary is concordant with the incomplete descriptions of these elements in the articles, reflecting the heterogeneity observed among studies. We were able to group these program elements broadly into 3 main categories: (1) program structure; (2) personnel; and (3) specific areas of counseling and/or education. All included reports incorporated at least 1 element from each category, the most common being a high-risk or prematurity clinic, coordination of care between all members of the team, a nurse case manager and/or care coordinator, nutrition counseling, and prenatal education. Of note, many programs did not distinguish between inclusion of patients at risk for medically indicated preterm birth (eg, secondary to preeclampsia) or spontaneous preterm birth (eg, secondary to spontaneous preterm labor). Medically indicated preterm birth is a competing outcome for spontaneous preterm birth, and the risk factors for preterm birth are similar regardless of indication. Further, some elements of case management, including increased surveillance and specially trained care-givers, may lead to earlier recognition of complications such as growth restriction and preeclampsia, leading to the potential for avoiding associated major complications. Therefore, “preterm birth” herein refers to both medically indicated and spontaneous preterm birth.

In general, case management programs address psychological, environmental, behavioral, and economic factors that are known or are thought to contribute to higher rates of preterm delivery. Programs commonly included a weekly phone call or regular home visits with the patient. However, we observed considerable variation among other program elements. For example, only 11 of the individual programs included smoking cessation programs despite consistent evidence of an association between cigarette smoking and preterm birth; at least 1 study reported a reduction in the rate of preterm birth after introduction of smoke-free legislation.^56^ Other elements that might contribute to the issue of avoidance or earlier detection of preterm delivery include the following: special prenatal clinics with more frequent visits and more specifically trained and focused providers and weekly phone calls and/or home visits. Additional components (eg, social worker, weekly phone call) increase the likelihood that patients will make it to their clinic visits and take prescribed medications regularly. Furthermore, programs such as smoking cessation and drug counseling address specific behaviors that are known to contribute to preterm birth. Psychology and social service counselors, also involved in a number of programs, have the potential for stress reduction and avoiding or decreasing the severity of other psychological disorders known to have a relationship with preterm birth.^57^ Given the diversity of the elements included in each program, it was not possible to distinguish which specific element(s) were the most beneficial.

In addition to variable program elements, the high-risk inclusion criteria for study enrollment varied among studies. This also likely impacted the ability to discern which individual elements conferred the greatest potential benefit (Table 2). When considering heterogeneity among studies, it is crucial to determine how each study assessed prematurity “risk”. Some risk assessment was relatively straightforward, such as including patients with a previous preterm birth^32^ or those receiving progesterone supplementation.^48^ In other cases, however, inclusion was broader and based on age (eg, teenagers),^32,47^ specific geographic areas of residence,^32,33,39,40,43–45,52^ race (eg, Black individuals),^35^ or insurer (such as a specific health insurance plan^37^ or Medicaid^20,42,51,53,55^). Still others used a telephonic or in-person survey screening tool for risk assessment and often did not define further how pregnancies were classified as high-risk for preterm birth.^30,31,33,34,44,55^

## Efficacy of case management programs

Evaluating whether case management programs are efficacious in preventing preterm birth was difficult owing to the heterogeneity among studies both with regard to program elements (Table 1) and participant inclusion criteria (Table 2). In addition, the study methodology, control populations, and outcomes chosen differed between programs.

Of the publications we reviewed, few involved randomized controlled studies; most were retrospective, observational cohort studies. Further, many studies included populations of patients before and after the intervention, whereas others used control groups that were similar geographically or consisted of patient populations sharing other common factors (such as the same insurance carrier or receiving care within the same health system). However, the populations receiving care varied even within the same health system, and it is likely that unmeasurable confounding factors affected program participation, precluding use of seemingly similar individuals as adequate controls. Outcomes evaluated across studies ranged from prematurity endpoints (eg, gestational age considered continuously vs absolute gestational age cutoffs) to neonatal outcomes (eg, birthweight, neonatal morbidities) to cost-related outcomes (eg, neonatal intensive care unit [NICU] admission, duration of stay, and cost of neonatal care).

In 2011, Whitworth et al^22^ performed a review of randomized trials of specialized prematurity clinics for the Cochrane Database Systematic Reviews. This review was limited to singleton gestations and included 3 studies^58–60^ with a total of 3,400 pregnant patients. The authors concluded that these 3 studies contained insufficient data on “prespecified outcomes” and insufficient sample sizes to demonstrate benefit (pooled relative risk for the 3 studies, 0.87; 95% confidence interval, 0.69–1.08). Randomized studies included in the Cochrane Review are detailed as follows. In 1989, Main et al^58^ randomized 943 inner-city pregnant individuals at high risk of preterm birth to a program that included frequent prenatal visits and cervical exams, education regarding signs and symptoms of preterm labor, and a 24-hour hotline to ensure easy access to obstetrical care. The authors found no difference in prematurity-related outcomes among the higher-risk patients included (preterm birth incidence of 23.2% in the intervention arm vs 20.7% in the control arm). They speculated that the program’s lack of impact on prematurity may have resulted in part from reduced program adherence among subjects in the study population. In 1989, Iams and Johnson^59^ presented results from their randomized trial of 370 pregnant patients, wherein those who were randomized to the study group, as in the analysis by Main et al, received care in a specialized clinic with frequent cervical exams. They also received specific preterm labor symptom education. In contrast to Main et al, Iams and Johnson found that more patients randomized to the study group received tocolysis and subsequently delivered at term than did those who received standard care. The Whitworth et al analysis reported the preterm birth rate in the Iams et al study to be 13.2% (24/182) in the study group and 18.6% (35/188) in the control group (relative risk, 0.70; 95% confidence interval, 0.44–1.14). Hobel et al^60^ in 1994 published results of a randomized controlled trial of 1774 patients participating in specialized clinics, which included special education and increased frequency of prenatal visits. This study found a 19% reduction in the preterm birth rate; this did not reach statistical significance, but “when risk factors for preterm birth were taken into account,” the finding was statistically significant (reduction from 9.1% to 7.4%; odds ratio, 0.78; relative risk,1.04; one-sided *P* value=.045). The Cochrane Review concluded that there was no clear evidence of benefit in terms of reduction in preterm birth rates with case management among these 3 studies. However, this was a small and early metaanalysis in the evolution of case management study evaluation, and the point estimate lay in the direction of benefit without the power to test for significance.

We identified only 1 other randomized controlled trial not included in the Cochrane Review. In 2001, Brooten et al^29^ randomized 173 high-risk pregnant patients with well-described, common risk factors for preterm delivery. The control group received usual prenatal care for high-risk patients at an academic medical center, and the study group received home visits by trained nurses on alternate weeks in addition to the usual prenatal care for high-risk patients. Each trained nurse visit included psychological and nutritional counseling, referral to community resources, education of signs and symptoms of preterm labor, monitoring of clinic visits, and medication compliance and home environment assessment. Individuals randomized to the nurse home visit intervention group were less likely to be admitted for threatened preterm labor and had a lower rate of delivery before 37 weeks’ gestation (preterm birth rate 30.9% in the intervention arm vs 40.8% in the control arm with no reported *P* values). Based on our calculations using the data provided, this reduction did not achieve statistical significance (*P*=.15). However, a significant reduction in perinatal mortality was noted for those randomized to the intervention arm vs the control arm (2/94 [2.1%] vs 9/98 [9.2%]; *P*=.035). The nurse home visit intervention group was also associated with overall savings of $2.8 million even when the costs of the visits were considered.

Among the 4 other systematic reviews and 1 metaanalysis identified, there were mixed conclusions. Three systematic reviews^23–25^ concluded that the results of individual trials varied, with overall fewer than half showing benefit or “promise”; the fourth^26^ concluded that there was ample evidence to suggest integration of case management into prematurity prevention strategies. The metaanalysis, conducted by Fernandez Turienzo in 2015,^27^ included 15 trials involving 22,347 pregnancies and concluded that individuals receiving “alternate care models” were less likely to experience preterm birth (relative risk,0.84; 95% confidence interval, 0.74–0.96). Most of the systematic reviews and the metaanalysis reported challenges such as those we observed with study comparisons, again given the wide variation in program methodology, including referral criteria or criteria for inclusion, interventions offered, and outcomes compared.

Thus, it is difficult to conclude definitive benefit of case management programs for prematurity prevention, primarily, we feel, because of the heterogeneity of the various programs, populations included, and outcomes selected. However, the combination of suggestive trends in the metaanalysis data and randomized trials (2 of 4 showing some benefit) suggest that case management programs have the potential to reduce preterm delivery, its consequences, and/or associated costs. It is important to note that several of the aforementioned “negative” studies are decades old. The programs described in these studies did not include elements of more comprehensive case management programs that were developed and described later; this point also was made in the Cochrane review. In addition, new interventions for specific risk factors such as short cervix on endovaginal ultrasound or previous preterm birth were not incorporated into the prematurity clinic algorithm; only a few of the more recent studies included patients on progesterone, and none included patients on low-dosage aspirin.

## Case management: time to implement now?

Several arguments support more wide-spread implementation of case management programs or specialty clinics for prematurity prevention. The rates of preterm birth continue to rise, though modestly, in a disturbing trend, and overall rates remain unacceptably high— approximately 10.1% after peaking at 12.8% in 2006.^61,62^ Existing pharmacologic interventions are limited. The known benefit of progesterone for prematurity prevention is restricted to patients with previous preterm birth and those with ultrasound-identified short cervix, both of which taken together affect only a small minority of patients who ultimately deliver prematurely. One of the few practices that might have influenced the recent reduction in the rate of prematurity involved policies and campaigns championed by national organizations (ACOG, March of Dimes, and others) to avoid elective delivery before 39 weeks’ gestation and the reduction of twin pregnancies through single-embryo transfers for individuals conceiving via in vitro fertilization and other artificial reproductive technologies.^63^ Recent attention has been paid to the contributions of patient stress, social support, and socioeconomic factors in the development of preterm birth and other adverse pregnancy outcomes.

Studies evaluating the cost effectiveness of case management have concluded that if improved outcomes are shown, programs are also cost-effective.^30,34^ Cost effectiveness can be maximized by proper patient selection, as efficacy— and therefore cost savings—appears greatest in those with the highest a priori risk of preterm birth. As described above, case management programs have become commonplace in fields of medicine outside of obstetrics that require comprehensive patient care. Further, most large health insurance plans have implemented and provide coverage for such programs in these fields.^64^ Insurers have concluded that major cost savings can be achieved. More specifically, as it potentially relates to patients at high risk of preterm delivery, 83% of insurers use case management focused on their highest-cost patients.^64^

Previous case management programs reporting on older cohorts used variable ways to define those at “high-risk” of preterm birth. Table 2 describes several population characteristics of patients included in the individual studies reviewed. However, many of these studies provided only vague details regarding criteria that placed an individual at high risk. More recent advances in risk stratification have improved clinicians’ abilities to identify pregnant individuals who are most likely to deliver prematurely. Careful population selection with more clear criteria to define individuals at high risk of preterm birth in addition to consideration of more contemporaneous tools (both those that are established and widely used clinically and those with clinical promise) to risk-stratify patients (eg, cervical length screening, cervical cytokine levels, and serum proteomic assessment)^65–67^ may have the potential to improve outcomes in case management programs and enhance cost efficiency. Of the 29 original papers reviewed, 20 provided information regarding whether outcomes in the case management program were improved if the population was risk-stratified vs evaluation across the overall population. All but 1^53^ of the 17 reports that included risk-stratified patients^20,30,32–35,38,40,44,45,47,49–51,53,55^ who were at a higher risk of preterm birth demonstrated reduced rates of preterm birth, low birthweight, and/or NICU admissions with case management. In addition, 2 reports that evaluated cost found overall reduced costs with case management.^30,34^ In contrast, in the 3 studies that included only patients with Medicaid or those with lower socioeconomic status but no other risk stratification, there was no reduction in preterm birth or low birthweight.^36,49^ These findings emphasize the importance of risk stratifying on multiple factors, including medical and socioeconomic factors but not socioeconomic factors alone.

Together, these data have demonstrated that there not only is a need to use all “medical” tools available to address the critical problem of prematurity but there is also the possibility that now, more than ever, specialized prenatal care programs may provide critically needed social and emotional support to patients at the highest risk of preterm birth. In addition, case management programs are low-risk, as they do not involve costly, experimental, or understudied drug therapies.

Because case management is low-risk and has the potential to reduce prematurity-related costs, it is reasonable to consider implementation of comprehensive programs—or some of their elements if limited resources are available—with the evidence presented here. However, as we have outlined, the current data are imperfect, and additional, high-quality evidence likely will be necessary before case management will be adopted widely as a recommended strategy for prematurity prevention. Unfortunately, there currently exists no optimal study design, as considerable variability appears in the selection of patients for inclusion and the interventions included in case management programs. Owing to the heterogeneity of preterm birth and its underlying etiologies, it is unlikely that a “one-size-fits-all” approach will be the most effective. Hence, our proposed program, as outlined in the Figure, centers around a case coordinator who is employed to facilitate individual-specific interventions, provide education, serve as a liaison with clinical providers, etc. This proposed program addresses medical, social, and socioeconomic factors that are established risk factors for prematurity. Randomized studies to evaluate such a program may be difficult to design to comprehensively address all elements of case management, but they would permit resource optimization by providing personalized care and services. In addition, such studies may permit evaluation of individual interventions within the program to establish those most beneficial. Further, identifying risk factors associated with greatest reduction in preterm birth will help refine the patient population determined eligible for case management. In our opinion, patients with the highest a priori risk of preterm birth should be the focus of initial clinical and research efforts. Once clarity is achieved regarding the optimal interventions and components of comprehensive, personalized case management programs, they might be extended and adapted to include lower-risk patients.

## Conclusions

Case management is a low-risk and underrecognized yet potentially beneficial element that can reasonably be considered as a component of prematurity prevention strategies in the United States. Tracking contemporaneous outcomes from ongoing prematurity prevention centers, especially those with broadly inclusive case management programs, should be considered. Further, evaluation of individual program elements from these centers will enable refinement of the optimal formula for case management program implementation at other institutions. In addition, multidisciplinary partnerships supported by grant funding, statistical support, and other means may help facilitate these analyses, including determination of cost efficacy.

## Figures and Tables

**FIGURE:**
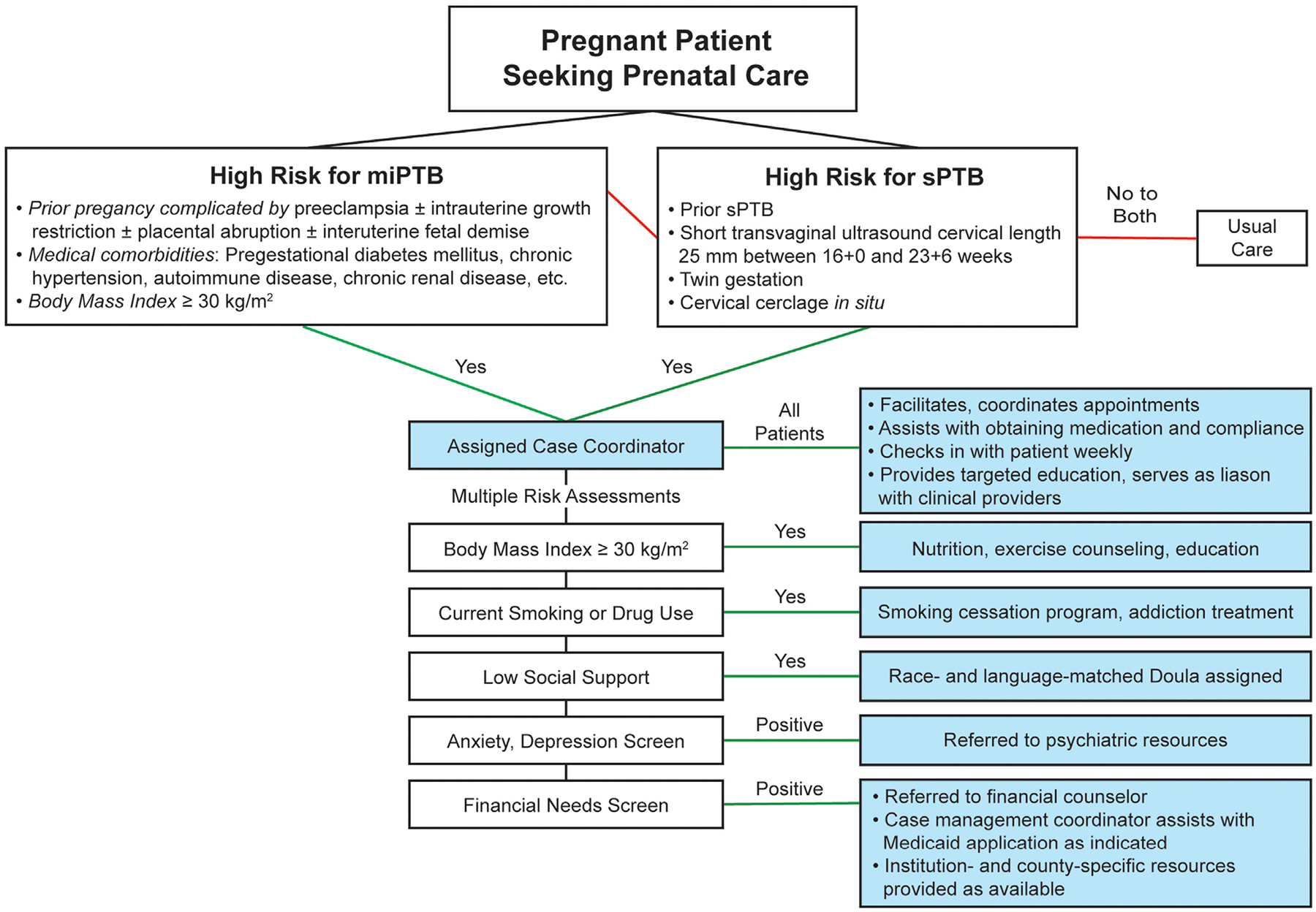
Proposed case management program structure *miPTB*, medically indicated preterm birth; *sPTB*, spontaneous preterm birth

**TABLE 1:** Elements of case management programs by broad type of program element

Program element		Number of studies
Program structure	Case manager phone calls and/or home visits	18
	Separate high-risk prenatal clinic	7
	Regular team conferences	3
Personnel	Case manager/program director	17
	Social worker	10
	Primary care provider as part of the team	5
	Nurse or certified nurse midwife coordinator	4
	Maternal-Fetal Medicine physician	3
Education or counseling	Prematurity education	19
	Nutrition counseling	14
	Smoking-cessation program	12
	Drug counseling	10

Listed are the number of studies, of 29 total reviewed, that included each component (almost all of them did not describe each element of case management and were often silent on whether any given element was included).

**TABLE 2:** Populations included in individual studies of the efficacy of case management

Populations	Number of studies
Nonspecific “high risk”	7
High risk of preterm delivery (previous preterm birth not specifically mentioned)^a^	6
Age <20 y at estimated date of confinement	2
Previous preterm delivery	3
Black race	1
Medicaid insurance or low socioeconomic status	4

a As defined by study authors; varied among studies
